# Assessment of Health Insurance Benefit Mandates for Fertility Preservation Among 11 US States

**DOI:** 10.1001/jamahealthforum.2021.4309

**Published:** 2021-12-03

**Authors:** Ricardo E. Flores Ortega, Sara W. Yoeun, Omar Mesina, Bonnie N. Kaiser, Sara B. McMenamin, H. Irene Su

**Affiliations:** Herbert Wertheim School of Public Health, University of California, San Diego, La Jolla; Herbert Wertheim School of Public Health, University of California, San Diego, La Jolla; Department of Obstetrics, Gynecology, and Reproductive Services, University of California, San Diego, La Jolla; Department of Anthropology and Global Health Program, University of California, San Diego, La Jolla; Herbert Wertheim School of Public Health, University of California, San Diego, La Jolla; Department of Obstetrics, Gynecology, and Reproductive Services, University of California, San Diego, La Jolla; Moores Cancer Center, University of California, San Diego, La Jolla

## Abstract

**IMPORTANCE:**

Multiple US states recently passed laws mandating health insurance coverage for fertility preservation (FP) services to improve access to care for patients with cancer, for whom FP service expenses can be prohibitive. Key unanswered questions include how heterogeneous benefit mandate laws and regulations are and how this variation may affect implementation, access, and utilization.

**OBJECTIVE:**

To describe the design of state-level FP health insurance benefit mandate laws and regulations and derive guidance on best practices and implementation needs.

**DESIGN, SETTING, AND POPULATION:**

Legal mapping and implementation science framework-guided analyses were conducted on 11 US state laws that mandate health insurance benefit coverage for FP services for patients at risk of iatrogenic infertility from medical treatments and on related insurer regulations. Design features of laws and regulations and the implementation process were summarized by themes (eg, coverage specification).

**EXPOSURES:**

State jurisdiction.

**MAIN OUTCOMES AND MEASURES:**

Main outcomes were the scope and specificity of mandated FP insurance coverage and the role of clinical practice guidelines and insurer regulations in implementation.

**RESULTS:**

Between June 2017 and March 2021, 11 states passed FP benefit mandate laws. States took a median (range) of 283 (0–640) days to implement mandates, and a majority issued regulatory guidance after the law was in effect. While standard-of-care procedures such as embryo cryopreservation require medical evaluation, medications, ultrasonography and laboratory monitoring, oocyte retrieval, embryo derivation, cryopreservation, and storage, there was variation in which services were specified for inclusion or exclusion in the laws and/or regulator guidance. The majority of state laws and regulator guidance reference medical society clinical practice guidelines and federal policies (Affordable Care Act and Health Insurance Portability and Accountability Act).

**CONCLUSIONS AND RELEVANCE:**

In this qualitative assessment of 11 state-level FP benefit mandates, variation that may influence patient access was identified in the design and implementation of the mandates. As clinical stakeholders aim to understand and/or shape these laws and their implementation, key considerations included specificity and flexibility of benefit design to be clinically meaningful, expansion of clinical practice guidelines to inform benefit coverage, inclusion of publicly insured and self-insured populations for universal access, and consistency between state and federal policies.

## Introduction

Nearly 70 000 people aged 0 to 39 years are diagnosed with cancer annually,^1,2^ and they can experience higher risks of infertility owing to cancer treatments. Infertility is a critical concern for many cancer survivors, but risks can be reduced by the evidence-based practice of fertility preservation (FP) care. However, the expense of standard FP services can be prohibitive and a common barrier to care. Thus, state-level health insurance benefit mandates that require health insurers to include coverage for FP services in specified types of health insurance plans have been passed in recent years to increase access and utilization of these services, but the effectiveness of this health policy intervention is unknown.^3–10^

Since 2017, 11 states (California, Colorado, Connecticut, Delaware, Illinois, Maryland, New Hampshire, New Jersey, New York, Rhode Island, and Utah) passed benefit mandates for insurance coverage of FP services, and more states have legislation under consideration.^11^ Reproductive and oncology health care professionals (physicians, advanced practice clinicians, nurses, social workers, financial counselors) and patients in states with benefit mandates have experienced barriers to and confusion around accessing FP insurance coverage.^12^ This suggests problems with benefit mandates and/or implementation at the state, insurer, clinic, or patient level. Benefit mandates are interpreted by state insurance regulators, which may issue guidance for implementation by insurers subject to the law. Insurers then design benefits that comply with the law and insurance regulator guidance and may inform contracting health care clinicians and patients of newly covered services. Finally, patients and clinics communicate with insurers to determine how to access benefits and understand terms of coverage for services. Key unanswered questions include how heterogeneous are benefit mandate laws and their regulation, and how might this variation influence implementation, access, and utilization. To date, commentaries have discussed these laws,^9,10,13^ but none have systematically analyzed them using a health policy and implementation lens to shape future legislation or inform implementation considerations by insurers and clinics.

The objectives of this state-level qualitative analysis are to systematically characterize variation in state-level FP health insurance benefit mandates and regulation using legal mapping^14^ and implementation science methods^15^ and to provide guidance on best practices, gaps, and implementation needs.

## Methods

### Data Set Development

In March 2021, a list of states (n = 11) that passed FP mandates was identified through the Alliance for FP, a nonprofit organization that tracks US FP legislation.^11^ Final versions of legislative text from state legislature websites were analyzed thematically.^16–26^ Additional searches were conducted on states’ insurance regulator website to identify FP legislation–specific guidance (n = 10 documents) generated by insurance regulators to insurers.^27–37^ Terms used in this search included *fertility preservation*, *all-plan letter*, *bulletin*, and *insurance check list*. Inquiries to each regulator confirmed that there were no other relevant communications. This research evaluated public documents; therefore, it was exempt from institutional review board review. This report followed the Consolidated Criteria for Reporting Qualitative Research (COREQ) reporting guidelines.

### Coding

Authors developed deductive (a priori) codes based on the Exploration, Preparation, Implementation, Sustainment (EPIS) framework (Figure).^15^ Implementation science frameworks guide systematic identification of strategies to improve delivery of evidence-based interventions. The EPIS framework was selected because it focuses on the innovation (benefit mandates), inner context (health insurer structure, culture, and leadership), outer context (insurance regulators, federal law, medical societies), and bidirectional bridging factors (insurance regulator guidance, clinical practice guidelines) within and between these contexts that influence implementation of the law. Initial review of legislative and regulator documents produced additional inductive codes (ie, arising from the data).

Using the preliminary codebook, 4 authors (R.F., S.Y., S.M., H.I.S.) coded legislative and regulator documents for 2 states. Coding discrepancies were reviewed, and codes were revised. Two coders (R.F., S.Y.) then independently coded all documents using MAXQDA 2020 (VERBI GmbH).^38^ Intercoder agreement exceeded 80%, and coding discrepancies were resolved through discussion.

### Data Analysis

Data were summarized by theme (eg, coverage specification [inductive], bridging factors [deductive]), with structured comparison of each theme across states.^39^ In legislative and regulator documents, references were made to medical society guidelines (American College of Obstetricians and Gynecologists [ACOG]; American Society of Clinical Oncology [ASCO]; American Society for Reproductive Medicine [ASRM]) and essential health benefit (EHB)–benchmark plans (plans in each state that determine what benefits are required to be covered by individual and small group plans). Guidelines from ACOG, ASRM, and ASCO were reviewed as needed to clarify legislative and/or regulatory language.^5,6,8^ Clarifying text was incorporated into theme summaries. The Centers for Medicare & Medicaid Services website was searched to provide context for the mention of EHBs in legislative and/or regulatory language.^40^

## Results

Between June 2017 and March 2021, 11 states passed FP benefit mandates (Table 1). Legislative text was obtained for all 11 states. Insurance regulator guidance was issued by all states except New Jersey and Utah. There were 2 regulator documents for New York. A total of 21 documents were analyzed.

### Policy Scan of Laws and Insurance Regulator Guidance

The enactment date (date the law was passed), effective date (date the law took effect), regulator communication date, insurance market segments affected by the FP benefit mandate, and preexisting in vitro fertilization benefit mandate are presented in Table 1. States took a median (range) of 283 (0–640) days to implement FP benefit mandates. Insurance regulators that issued guidance regarding implementation took a median (range) of 231 (95–637) days. Only 3 regulators issued communication prior to or on effective dates of mandates. Ten laws applied to commercial large group (ie, >50 employees) plans, with variation in requirements for individual and small group plans. Illinois was the only state with coverage applicable to both commercial and state Medicaid plans, while Utah’s law only applied to Medicaid plans.

### How Is Iatrogenic Infertility Defined?

Ten states’ laws (excluding Utah) and/or regulator guidance (excluding New Jersey) included a definition for iatrogenic infertility as infertility arising from treatments that directly or indirectly cause infertility. Most states (excluding Maryland and Delaware) cited not only cancer chemotherapy, radiation therapy, and surgery but also broadened to other treatments that could cause infertility. No laws were more specific on eligible treatments (eg, chemotherapy regimen or radiation site). Five states (California, Maryland, New Jersey, New York, Rhode Island) referred to medical societies to set standards for medical treatments that can cause infertility.

### What FP Services Are Covered?

Standard-of-care FP services such as embryo cryopreservation require medical evaluation, medications, ultrasonography and laboratory monitoring, oocyte retrieval, cryopreservation, and storage. There was variation in which services were specified for health insurance inclusion or exclusion (Table 2). Delaware law included the broadest scope of specified covered services. In contrast, California law did not define coverage for any specific services. Whereas oocyte and sperm freezing were specified in 6 states, embryo freezing was specified in only 4 states. Four states specified coverage for storage, while 3 specified exclusion of storage. Four states set limits on total costs or number of cycles. All states except Connecticut referred to clinical guidelines to set what FP procedures are considered standard of care, allowing for coverage of future standard-of-care procedures that may develop over time.

### Additional Limitations and Prevention of Restrictions

Four states allowed religious employers to request exemptions for coverage. Most states did not specify patient age restrictions on eligibility, except for Delaware law (oocyte retrievals before age 45 years), Illinois regulator guidance (age 14–45 years), and New York law (age 21–44 years). While New York legislation included age limitations, guidance that followed from the state insurance regulator indicated that age restrictions are not permitted. Three states specified that coverage cannot be restricted based on life expectancy or predicted disability.

### Cost-Sharing

Cost-sharing (out-of-pocket costs for patients) was described by 7 states. These states allowed copayments, coinsurance, deductibles, and benefit maximums. Five states included parity language (language that requires benefits to be covered for FP services similarly to non-FP services) to prevent insurers from charging more via deductibles, copayments, and/or coinsurance) or putting more restrictions on FP benefits (eg, benefit maximums and waiting periods) that are not in place for other, non-FP medical services (eg, cancer surgery). In contrast, Connecticut regulator guidance allowed insurers to apply plan-level cost-sharing mechanisms and coinsurance of up to 50%. Connecticut was the only state that explicitly allowed prior authorization. For both Connecticut and New York, cost-sharing was subject to insurance regulator oversight.

### Implementation of Benefit Mandates

The EPIS framework was used to describe key themes in the documents that could influence benefit mandate implementation (Figure). Within EPIS, benefit mandate legislation is implemented in the inner context by the health insurer and is influenced by related state and federal health insurance policies and medical societies in the outer context, as well as by bridging factors between the inner and outer contexts (eg, regulator interpretation and communication). No inner context themes (ie, how health insurer structure, culture, or leadership influenced implementation of benefit mandates) were identified; outer context and bridging factor themes are described below.

#### Outer Context

Federal laws, such as the Affordable Care Act (ACA) and the Health Insurance Portability and Accountability Act (HIPAA), influence state-level benefit mandates. The ACA established a mechanism for determining EHBs requiring coverage in nongrandfathered individual and small group market plans. States are required to defray costs of providing benefits if the benefit mandate exceeded the state-defined EHB-benchmark plan,^41^ making it difficult to pass benefit mandate legislation if it is interpreted to exceed EHBs. Colorado and California laws made specific arguments that FP mandates do not exceed EHBs because they are covered under an existing pregnancy benefit (Colorado) or are already covered as a basic health care service (California). Colorado and Illinois included language that would render the mandates inoperable should the federal government determine that FP services exceed EHBs.

In accordance with section 1557 of the ACA, Connecticut insurance guidance clarified that age limits are discriminatory if applied to services that are clinically effective at nonincluded ages. In Connecticut and New York insurance regulator guidance, lifetime limits on FP benefits were determined to be an “impermissible preexisting condition exclusion” under HIPAA.

#### Bridging Factors

Bridging factors centered on communication across levels. Explicit reference to clinical guidelines in legislation or regulator guidance bridges medical societies with insurers. Laws in 10 states referred to ACOG, ASRM, and/or ASCO guidelines to set procedures and services consistent with standard-of-care for FP (Table 3). Five states (California, Maryland, New Jersey, New York, and Rhode Island) additionally referred to clinical societies’ standards for medical treatments that can cause infertility.

Several themes within regulator guidance demonstrated how these documents serve to influence implementation as bridging factors between regulators and insurers. Four states’ regulator guidance added coverage details beyond legislation (Table 4). For example, New York regulator guidance clarified that reproductive tissue storage is a covered benefit. Second, discordance between mandate benefit legislation and federal regulations was noted in multiple regulator documents. For example, Connecticut guidance instructed insurers to remove age limits on FP benefits. Finally, guidance specified for insurers to inform insured members on the benefit and communicate with regulators for compliance. In California, the insurance regulator instructed insurers to review and modify a range of consumer-facing documents (eg, Summary of Benefits and Evidence of Coverage) to ensure compliance.

## Discussion

Substantial prior and ongoing efforts to support health insurance coverage of FP services have been followed by state-level insurance benefit mandates. These mandates display heterogeneity that may influence implementation and, ultimately, patients’ access to care. This work highlights the importance of systematic evaluation of health policies to guide best practices, note gaps, and identify implementation needs. We characterized variation in benefit mandate design and implementation time frame and documented how benefit mandates are influenced by external factors and, in turn, how regulator communication and clinical practice guidelines are central to implementation of benefit mandates by health insurers.

### Benefit Mandate Design

Several best practices for FP benefit mandate design are proposed, informed by study findings. First, legislative language needs to be specific yet flexible, such that the scope of services to be covered is not up to interpretation by health insurers but is responsive to needs of individual patients. Nonspecific language leads to heterogeneity, a patchwork of benefits, and potentially a more restricted scope of coverage defined by insurers, which may not include important benefits, such as in vitro fertilization and storage of cryopreserved material. Language specifying flexibility regarding the number of FP cycles would ensure that coverage is robust and clinically meaningful; however, this was not observed in most legislation to date and would benefit from clinical stakeholder input.

Second, FP coverage needs to be universally available. Fertility preservation is largely available to those who can pay the high costs. Only 2 states included Medicaid beneficiaries under their mandate. While basic Medicaid benefits are set at the federal level, states have the option of mandating additional benefits to their Medicaid enrollees that are not mandated at the federal level. Interestingly, the Utah law applies to Medicaid but not commercial payers. Ensuring health equity requires inclusion of publicly insured and self-insured populations in FP benefit mandates and necessitates continued clinical advocacy.

Finally, legislation should, but often did not, include language that establishes FP benefits at parity with other non-FP covered health care services (eg, cancer surgery). Ensuring parity results in treating all individuals who need FP services fairly, by preventing insurers from charging more or putting greater restrictions on FP benefits compared with non–FP-related medical care.

### Implementation Time Frame

We observed substantial variation (up to 2 years) in the implementation period between laws’ enactment dates and corresponding effective dates. It is crucial to allow adequate time for insurance regulators to generate guidance and for insurers to make modifications to their standard benefit plans and communicate changes to contracting clinicians and enrollees. Ideal implementation periods likely vary by context/resources for implementation and should be informed by empirical data, which are currently lacking. Only 1 state (Colorado) had legislation-appropriated funds ($3337) for implementation; other states should follow suit.

### Outer Context Constraints and Facilitators

Outer context constraints include state and federal health insurance policy. Processes for states to select EHB-benchmark plans enable FP benefits and infertility benefits to be defined as an EHB. This is important because mandated benefits that exceed state-defined EHBs post an additional cost to states. Of the 11 states, 8 included coverage for infertility services in their 2017 EHB-benchmark plan.^40^ While no state currently includes explicit coverage for FP in its benchmark plan, it is possible to argue that FP mandates do not exceed EHBs because they clarify existing covered infertility benefits. Arguing how proposed FP benefit mandates do not exceed EHBs is an essential strategy for political viability of legislation. In addition, by leveraging ACA and HIPAA antidiscrimination laws, advocates can push back against language that requires lifetime or age limitations.

### Bridging Factors as Facilitators

Importantly, through clinical practice guidelines, medical societies that set care standards based on clinical expertise and best evidence can have major influence on health policy interventions. National clinical practice guidelines enable recategorization of experimental to standard-of-care procedures over time (eg, ovarian tissue freezing) that may then be included in benefits for standard-of-care FP services without each state specifying a new service. With little language specifying the types and number of services to be covered as standard of care within present-day laws and regulator guidance (Table 3), there is an opportunity for medical societies to revise current guidelines to expand coverage to meet the breadth of needs and indications in patients. For example, if state law requires coverage of oocyte cryopreservation but not related medications, oocyte retrieval, and storage, the cost of fertility preservation may continue to be unaffordable for most patients. As well, many laws refer to ASRM or ASCO guidelines to define the at-risk population, but only ACOG guidelines currently specify which treatments may cause infertility. The ASRM and ASCO clinical guidelines should encompass evidence-based infertility risk stratification, as well as recommendations for management where treatment-related infertility risks are unknown. Although better-defined at-risk populations in guidelines may narrow the population of eligible patients, the status quo leaves room for broad or narrow interpretations by insurers and discretion on rules and processes that define medical necessity.^42^ For example, a woman who will undergo a hysterectomy may not be considered to be at risk of iatrogenic infertility by some insurers if she still retains her ovaries. Until referenced guidelines define at-risk populations, legislators should include language designating oncology and fertility medical professionals to make the clinical decision.

Insurance regulators are a key player in benefit mandate implementation. The EPIS bridging factors construct enabled characterization of how regulators bridged mandates with state and federal policies and health insurance plans. This policy scan showed that regulator guidance varied in scope and frequently highlighted how state legislation conflicted with federal law (eg, age restrictions and lifetime limitations). More than half of the regulators issued guidance related to implementation of the FP mandate after the mandate went into effect. To have a meaningful influence on the implementation process, regulator guidance should be issued prior to the mandate effective date. Regulator communication may be influenced by stakeholder feedback and presents an opportunity to ensure compliance with existing laws and to specify and update scope of coverage. More research on processes that insurance regulators undertake to shape their guidance documents is needed to inform interventions on regulators.

The strength of this work centers on informing a clinical audience on applying health policy and implementation science perspectives to laws that influence the practice of medicine, not only to improve understanding of complexities of health insurance laws but also to identify points where clinical stakeholders can intervene. This work also augments the few existing commentaries by including insurance regulator guidance to complement laws and updating current FP benefit mandates with a focus on variation among states.^9,10,13^

### Limitations

The major limitation of this work is a lack of systematically collected insurer-level, clinic-level, and patient-level data on benefit mandate implementation, which is needed to identify determinants, moderators, and mediators of patient utilization of FP benefits within benefit mandates and insurance regulator guidance. These data would allow comparison of implementation and utilization data by state law and insurance regulator guidance. Finally, it is possible that regulators issued additional guidance related to FP benefit mandate implementation after the last search was conducted (October 2021).

## Conclusions

This policy scan documented variation in design and implementation of health insurance benefit mandates for FP services across 11 states with enacted laws. As clinical stakeholders aim to understand and/or shape these laws and their implementation, key considerations include specificity and flexibility of benefit design to be clinically meaningful, expansion of clinical practice guidelines to inform benefit coverage, inclusion of publicly insured populations in required coverage, consistency with relevant state and federal policies, sufficient lengths of time for the implementation period, timely regulator guidance issued to facilitate implementation, supportive medical society guidelines, and explicit allocation of resources for the implementation process.

## Figures and Tables

**Figure.**
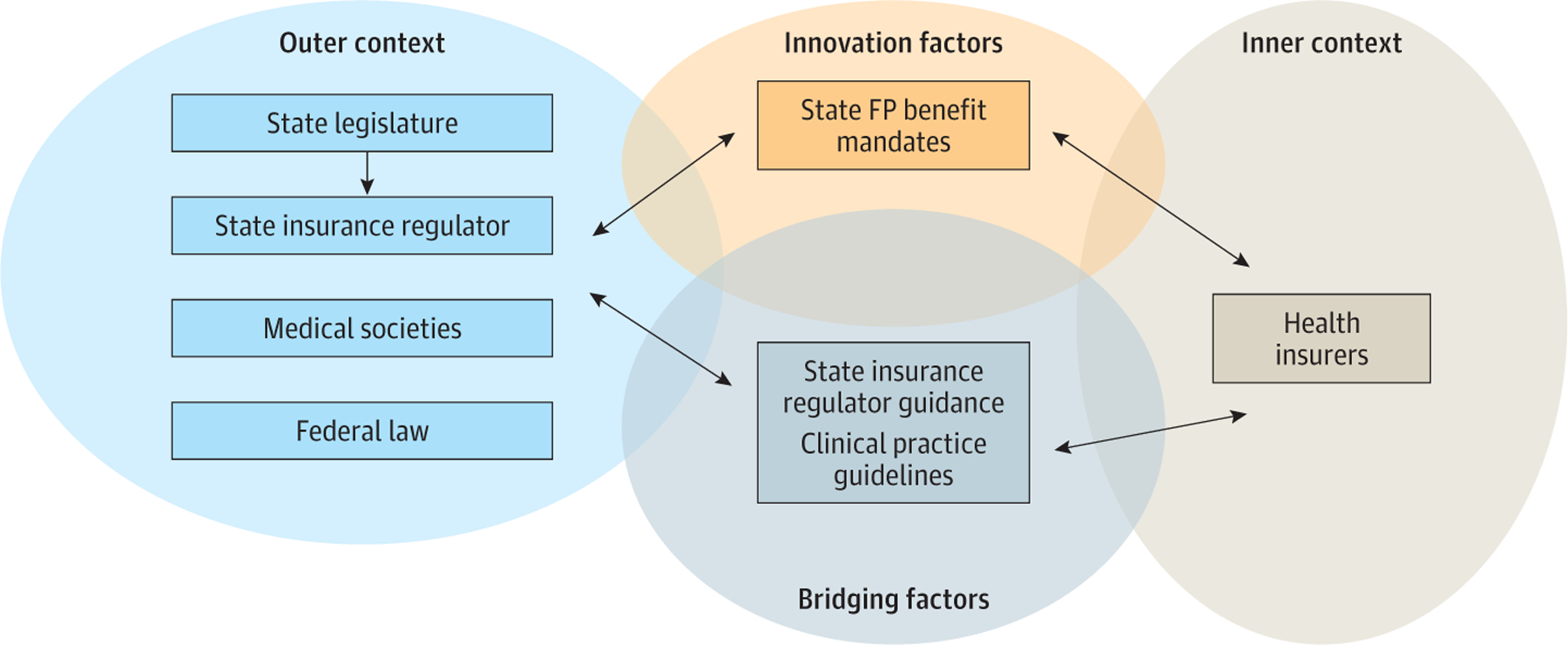
Key Domains in Fertility Preservation (FP) Mandate Implementation Exploration, Preparation, Implementation, Sustainment (EPIS) framework guided the systematic evaluation of key domains (innovation, outer context, inner context, bridging factors) known to influence downstream implementation.

**Table 1.** Summary of Fertility Preservation Benefit Mandate Enactment, Insurance Regulator Communication, and Affected Insurance Market Segments by State

State	Benefit mandate enactment date	Benefit mandate effective date	Regulator communication date	Days between enactment date and		Commercial market plans			Public plans	Preexisting IVF benefit mandate
				Effective date	Regulator communication date	Individual	Small group^a^	Large group	Medicaid	
California	10/12/2019	10/12/2019^b^	01/15/2020	0	95	Included	Included	Included	Not included	No
Colorado	04/01/2020	01/01/2022	07/30/2020	640	120	Included	Included	Included	Not included	No
Connecticut^c^	06/20/2017	01/01/2018	03/19/2019	195	637	Not included	Included	Included	Not included	Yes
Delaware	06/30/2018	06/30/2018	10/09/2018	0	101	Included	Not included	Included	Not included	Yes
Illinois	08/27/2018	01/01/2019	05/01/2020	127	613	Included	Included	Included	Included	Yes
Maryland	05/15/2018	01/01/2019	01/01/2019	231	231	Not included	Not included	Included	Not included	Yes
New Hampshire	08/05/2018	01/01/2020	04/13/2020	514	617	Not included	Included	Included	Not included	No
New Jersey	01/13/2020	04/12/2020	NA	90	NA	Not included	Included	Included	Not included	Yes
New York^c^	01/13/2019	01/01/2020	12/03/2019	353	324	Included	Included	Included	Not included	No
Rhode Island	07/05/2017	07/05/2018	12/08/2017	365	156	Included	Included	Included	Not included	Yes
Utah^d^	05/16/2021	01/01/2023	NA	595	NA	Not included	Not included	Not included	Included	No

Abbreviations: IVF, in vitro fertilization; NA, not applicable.

a Defined as 50 or fewer full-time equivalent employees.

b Policy enacted in 2019 clarified that existing law already required coverage of fertility preservation services as part of “basic health care services.”

c Coverage limited to individuals who previously maintained coverage under the policy for at least 12 months.

d Pending Centers for Medicare & Medicaid Services approval.

**Table 2.** Summary of Variation in Health Insurance Benefit Mandate Coverage Specifics for Fertility Preservation (FP) for Iatrogenic Infertility by State

	California	Colorado	Connecticut	Delaware	Illinois	Maryland	New Hampshire	New Jersey	New York	Rhode Island	Utah
ASRM, ASCO, or ACOG guideline-based standard-of-care FP services	Included	Included	NS	Included	Included	Included	Included	Included	Included	Included	Included
Services associated with FP (medical evaluation, ultrasonography, medication, laboratory work)	NS	Included	NS	Included	Included	Included	Included	NS	Included	NS	NS
Oocyte retrieval	NS	Included	Included	Included	Included	Included	Included	NS	Included	NS	NS
Oocyte cryopreservation	NS	NS	NS	Included	Included	Included	Included	NS	Included	NS	Included
Embryo cryopreservation	NS	NS	NS	Included	NS	NS	Included	NS	Included	NS	Included
Sperm cryopreservation	NS	NS	NS	Included	Included	Included	Included	NS	Included	NS	Included
Other reproductive tissues cryopreservation	NS	NS	NS	Included	NS	NS	Included^a^	NS	Included	NS	Included
Storage	NS	NS	Excluded	Included	Included	Excluded	Optional	Excluded	Included	NS	Included
Cycle limitations	NS	3 Oocyte retrievals	2 IVF cycles^b^	6 Oocyte retrievals	NS	NS	NS	NS^c^	3 IVF cycles	$100 000^d^	NS
Experimental FP procedures	NS	NS	NS	Excluded	NS	NS	Excluded	NS	Excluded	NS	Excluded

Abbreviations: ACOG, American College of Obstetricians and Gynecologists;

ASCO, American Society of Clinical Oncology; ASRM, American Society for Reproductive Medicine; IVF, in vitro fertilization; NS, not specified.

a So long as they are not determined to be an experimental infertility procedure.

b Connecticut legislation lifetime limits of 2 IVF cycles was overwritten by the Connecticut regulator.

c New Jersey legislation lifetime limit of 4 oocyte retrievals was overwritten by the New Jersey regulator.

d Lifetime cap per insurance contract.

**Table 3.** Clinical Guideline Summaries and Comparison

	ASCO^5^	ASRM^6^	ACOG^8^
Target audience	Oncology health care clinicians	Reproductive medicine health care clinicians	Gynecology health care clinicians
Target patient population	Patients with cancer at risk for infertility owing to anticancer treatment	Patients undergoing gonadotoxic therapy or gonadectomy	Female patients with cancer diagnosed at age 20 y and younger
Specification of which patients are at risk for infertility or what constitutes gonadotoxic therapy	NS	NS	Hypothalamic or pituitary irradiation ≥30 Gy
			Ovarian–uterine irradiation ≥5 Gy
			Increased use of alkylating agents
Recommendation for oncology health care clinicians to address the possibility of infertility and refer interested patients to reproductive specialists	Yes	Yes	Yes
Recommendation for counseling on risks of genetic damage to sperm after chemotherapy	Yes	NS	NS
Recommendation to discuss FP success rates, FP alternatives (eg, donor gametes), need for gestational surrogacy	NS	Yes	NS
Recommendation on quantity of FP procedures (eg, number of sperm collections, oocyte retrievals, laboratory services, ultrasounds)	NS	NS	NS
Recommendation to discuss FP costs	Yes	Yes	NS
Recommendation to discuss additional gynecologic issues, including contraception, sexuality, pubertal development	NS	NS	Yes
Postpubertal male standard-of-care FP procedures: sperm cryopreservation	Yes	Yes	NS
Postpubertal male experimental FP procedures: testicular tissue cryopreservation and reimplantation	Yes	NS	NS
Postpubertal female standard-of-care FP procedures			
Embryo cryopreservation	Yes	Yes	Yes
Oocyte cryopreservation	Yes	Yes	Yes
Ovarian transposition	Yes	Yes	Yes
Ovarian suppression	Yes, in patients with breast cancer	Not standard of care	Yes, in postpubertal females
Ovarian tissue cryopreservation	Not standard of care	Yes	Yes
Prepubertal male testicular tissue cryopreservation	Experimental	Experimental	NS
Prepubertal female ovarian tissue cryopreservation	Experimental	Standard of care	NS
Long-term storage of cryopreserved tissue and gametes as standard of care	NS	NS	NS
Clinical FP program requirements (rapid access, interdisciplinary team, assisted reproductive technology laboratory, mental health, genetic and financial counselors)	NS	Yes	NS

Abbreviations: ACOG, American College of Obstetricians and Gynecologists; ASCO, American Society of Clinical Oncology; ASRM, American Society for Reproductive Medicine; FP, fertility preservation; NS, not specified.

**Table 4.** Comparison of Benefit Mandate Statute and Insurance Regulator Guidance^a^

	Guidance does not augment statute	Guidance augmented statute	Guidance disputed/restricted statute	Not addressed in either document
Coverage specifics	California, Colorado, Delaware, Maryland, Rhode Island	Connecticut, Illinois, New Hampshire, New York	None	None
Cost-sharing	Colorado, Delaware, New Hampshire, New York, Rhode Island	Connecticut, Maryland	None	California, Illinois
Enactment date	California, Colorado, Connecticut, Maryland, New Hampshire, New York, Rhode Island	Delaware, Illinois	None	None
FP services as an essential health benefit	Illinois, Maryland	Colorado	None	California, Connecticut, Delaware, New Hampshire, New York, Rhode Island
ACOG, ASRM, or ASCO guideline reference	California, Colorado, Delaware, Illinois, Maryland, New Hampshire, New York, Rhode Island	None	None	Connecticut
Age restriction/lifetime limits	Colorado, Delaware, Rhode Island	None	Connecticut, Illinois, New York	California, Maryland, New Hampshire
Religious organization exemption	Colorado, Connecticut, Delaware, Maryland	None	None	California, Illinois, New Hampshire, New York, Rhode Island
Insurance markets	Colorado, Connecticut, Delaware, New Hampshire, Rhode Island	California, Illinois, Maryland, New York	None	None
Parity	Colorado, Delaware, New Hampshire, New York	None	None	California, Connecticut, Illinois, Maryland, Rhode Island
Defines iatrogenic infertility	California, Colorado, Delaware, Illinois, Maryland, New Hampshire, New York, Rhode Island	Connecticut	None	None
Communication with beneficiaries	Connecticut, Delaware	California	None	Colorado, Illinois, Maryland, New Hampshire, New York
Appropriation for implementation	Colorado	None	None	California, Connecticut, Delaware, Illinois, Maryland, New Hampshire, New York, Rhode Island

Abbreviations: ACOG, American College of Obstetricians and Gynecologists; ASCO, American Society of Clinical Oncology; ASRM, American Society for Reproductive Medicine; FP, fertility preservation.

a Excludes New Jersey and Utah, which did not have insurance regulator guidance during the study period.
